# Unraveling the complexity of PRRSV-2 recombination: recombination frequency, replication efficiency, and backbone fitness define highly recombinogenic backbone and evolutionary bottlenecks

**DOI:** 10.1128/spectrum.01184-26

**Published:** 2026-07-17

**Authors:** Weixin Wu, Xinyu Fang, Xiang Gao, Junnan Zhang, Qiongqiong Zhou, Peng Gao, Yongning Zhang, Xinna Ge, Jun Han, Xin Guo, Lei Zhou, Hanchun Yang

**Affiliations:** 1 State Key Laboratory of Veterinary Public Health Safety, College of Veterinary Medicine, China Agricultural University 34752, Beijing, China; 2 Key Laboratory of Animal Epidemiology of Ministry of Agriculture and Rural Affairs, College of Veterinary Medicine, China Agricultural University 34752, Beijing, China; 3 National Engineering Laboratory for Animal Breeding and Key Laboratory of Animal Genetics, Breeding and Reproduction, Ministry of Agriculture and Rural Affairs, College of Animal Science and Technology, China Agricultural University 34752, Beijing, China; Changchun Veterinary Research Institute, Chinese Academy of Agricultural Sciences, Changchun, China

**Keywords:** porcine reproductive and respiratory syndrome virus, NADC30-like, vaccine, recombination

## Abstract

Recombination is a primary driver of porcine reproductive and respiratory syndrome virus (PRRSV) evolution. Currently, lineage 1 NADC30-like virus (L1C) and lineage 8 HP-PRRSV (L8E) recombinants dominate in China, whereas recombinants harboring the lineage five backbone are infrequently detected. To elucidate the mechanisms underlying this disparity, we investigated the interplay between recombination frequency, viral fitness, and strain-specific phenotypes using co-infection models. Next-generation sequencing (NGS) revealed that the lineage 5 replicase is prone to template switching, generating high frequencies of recombination junctions comparable to NADC30-like strains when viral RNA abundance is high. However, plaque purification demonstrated a disconnection between the generation of recombination events and the survival of viable progeny. While NADC30-like strains acted as a highly recombinogenic backbone, yielding up to 100% recombinant progeny, lineage 5-based recombinants were largely eliminated due to competitive exclusion. Furthermore, although a mutation known to reduce recombination frequency (K541R) successfully reduced errors at the nucleic acid level, it failed to prevent the emergence of recombinants under strong selective pressure. Our findings suggest that PRRSV recombination is not merely a stochastic event but a complex outcome dictated by strain-specific phenotypic profiles. The scarcity of lineage 5 recombinants is driven by a phenotypic deficit, which is a combination of replication efficiency and susceptibility to host clearance. This study highlights the dominant role of NADC30-like strains in driving viral diversity and underscores that vaccine safety depends on the holistic phenotypic fitness of the virus.

**IMPORTANCE:**

Porcine reproductive and respiratory syndrome virus (PRRSV) recombination poses a significant threat to the farm as it accelerates viral evolution and leads to changes in pathogenicity and immune protection. Understanding why certain strains recombine frequently while others do not is critical for reducing this risk. This study unravels the complexity of PRRSV recombination, showing that it is determined by the specific phenotypic profile of the virus strains involved. We discovered that current epidemic strains (NADC30-like) act as highly recombinogenic backbones due to their biological traits. Surprisingly, classical vaccine strains (lineage 5) infrequently form viable recombinants not because they are genetically accurate, but because their distinct phenotypic weaknesses lead to their elimination by the host or competitors. This finding shifts the paradigm of vaccine safety, emphasizing that preventing recombination requires a holistic approach that considers the complex interplay of viral fitness and host adaptation, thereby providing critical guidance for the industry.

## INTRODUCTION

Porcine reproductive and respiratory syndrome (PRRS), caused by the PRRS virus (PRRSV), poses a significant economic loss to the global swine industry. Highly pathogenic PRRSV (HP-PRRSV) infections can result in mortality rates of up to 100% in pigs (1, 2). According to the International Committee on Taxonomy of Viruses (ICTV) Master Species List #40 (v2, August 2025), PRRSV is classified into *Betaarterivirus europensis* (also known as PRRSV-1) and *Betaarterivirus americense* (also known as PRRSV-2) within the genus *Betaarterivirus* (https://ictv.global/taxonomy/). The PRRSV genome is an approximately 15-kb positive-sense single-stranded RNA molecule containing at least 10 identified open reading frames (ORFs) (3, 4). ORFs 2–7 encode structural proteins (SPs), which are crucial for inducing homologous immunity (5–10). ORF1a and ORF1b encode viral replicase polyproteins pp1a and pp1ab, which are processed into at least 12 nonstructural proteins (nsps) involved in transcription and replication (11, 12). Among these, nsp9 contains the RNA-dependent RNA polymerase (RdRP) domain, serving as the core component of the virus replication-transcription complex (RTC) (13).

PRRSV-2 is classified into 11 genetic lineages (L1–L11) and 21 sublineages (L1A‒L1F, L1H‒L1J, L5A‒L5B, L8A‒L8E, and L9A‒L9E) based on ORF5 sequences (14). In China, lineages 1, 3, 5, 7, 8, and 9 have been detected, with lineages 1, 3, 5, and 8 being the most prevalent (14, 15). Since being introduced around the year 2013, lineage 1 NADC30-like strains (L1C) have circulated in China for over a decade (16, 17), and become the dominant epidemic strains (18–20). A similar epidemiological dominance of L1C strains is also observed in the United States (14). Lineage 5 strains generally retain ancestral genomic features and lack key virulence-enhancing mutations present in modern epidemic strains, making them more susceptible to host clearance mechanisms (21, 22). Lineage 5 wild-type strains have largely disappeared from the field in recent years. Current detections of lineage 5 in China are primarily associated with the use of modified live vaccines (MLV) rather than naturally circulating wild-type viruses (14). Lineage 8 HP-PRRSV (L8E) emerged in China in 2006 (1), after which several HP-PRRSV-derived MLV vaccines were commercialized and widely used (23). Reports of wild-type HP-PRRSV in the field have been absent in recent years, whereas MLV-derived strains continued to reappear (24, 25). As an RNA virus, PRRSV exhibits high mutation and recombination rates, making its epidemiology highly complex. Traditional classification based solely on ORF5 cannot reveal the recombination property of field strains (26). Whole genome sequencing (WGS) surveillance has revealed a yearly increase in NADC30-like recombinant strains, particularly those recombining with lineage 8 HP-PRRSV, while non-recombinant strains are actually reduced in proportion (20, 27). Furthermore, specific nucleotide mutations indicate that the lineage 8 strains involved in recombination events were predominantly derived from lineage eight vaccines (28). Interestingly, while lineage 1 and lineage 8 strains are frequently involved in recombination events, recombinants involving lineage 5 strains are infrequently detected in the Chinese field (27). This observation is notable because classical lineage five passage-attenuated strains are widely utilized as vaccines in pig farms, meaning the vaccine strain itself is present in most vaccinated herds. Whether this discrepancy is due to differences in recombination frequency or viral fitness remains unclear.

Recombination in PRRSV is traditionally described as a “copy-choice” mechanism (29, 30), where the RdRP switches templates during synthesis (29). However, recent studies suggest that this process is more dynamic than a simple precise switch. Recombination has been increasingly viewed as a fitness-driven, iterative process that often begins with “imprecise” intermediates, at times independent of sequence identity (31, 32). Furthermore, a “forced-copy-choice” mechanism has been identified, wherein the polymerase adds non-templated nucleotides to facilitate template switching, effectively serving as an RNA repair mechanism to rescue damaged genomes (33). Together, these findings underscore that viral replication accuracy, which refers to the precision with which the replicase navigates these templates (34), is a key factor in this process. Our previous study identified the nsp9-10 region as a critical determinant of PRRSV accuracy. We found that the NADC30-like strain CHsx1401 exhibits lower accuracy and higher frequency of self-recombination (intra-recombination) compared with the HP-PRRSV strain JXwn06 (35). Furthermore, a conserved key residue, nsp9 K541, located on the template RNA channel, was confirmed to be a key factor that influences PRRSV replication accuracy. The K541R mutation was shown to reduce recombination frequency, consequently reducing self-recombination events in JXwn06 (36).

However, previous studies focused primarily on single-strain infection. There is a lack of research concentrating on inter-strain recombination under co-infection, a scenario where two or more viruses infect the same host cell (29). Recombination outcomes in such complex scenarios are not determined by a single factor but are likely the result of a multi-dimensional interplay involving recombination frequency, replication efficiency, viral productivity, host selective pressure (37), and superinfection exclusion (38). Distinct PRRSV lineages exhibit widely varying phenotypic traits, yet how these strain-specific differences interact to shape recombination landscapes remains largely unknown. It remains unclear whether the high prevalence of NADC30-like and HP-PRRSV recombinants is driven by replicase errors or other ecological advantages. Concurrently, the underlying reason for the scarcity of lineage 5 recombinants is also unclear, with open questions centering on whether this is attributable to low recombination frequency or the inability of their progeny to survive host selection. Furthermore, it is yet to be determined if reducing recombination frequency in vaccine strains can effectively mitigate the generation of novel recombinants in a clinical setting.

To address these questions, we established a co-infection model using CHsx1401 (representing lineage 1 NADC30-like strain), BJ-4 (representing lineage five classical strain), and JXwn06 P80 (representing lineage 8 HP-PRRSV vaccine strain). Additionally, to explore whether the K541R mutation could improve the safety profile of live attenuated vaccines by limiting recombination, we introduced the K541R mutation into a previously constructed chimeric vaccine candidate, RvBJ-4-(ORF2-6)_SX_ (5). This chimera contains the ORF2 to ORF6 coding structural proteins of CHsx1401 (for homologous protection) within a BJ-4 backbone (hypothesized to be safer). By combining next-generation sequencing (NGS) with plaque purification and WGS, we revealed a disconnect between nucleic acid recombination and viable virus isolation. While the K541R mutation reduced recombination frequencies at the RNA level, it failed to prevent the emergence of recombinants under the strong selection pressure exerted by NADC30-like or HP-PRRSV strains. Our work demonstrates that PRRSV recombination is a complex process dictated by the distinct phenotypic profiles of the participating strains. We show that while intrinsic template-switching propensity contributes to the generation of diversity, the ultimate survival of recombinants is determined by a rigorous selection for specific phenotypic traits, such as backbone fitness and replication efficiency.

## RESULTS

### NADC30-like recombinants are the predominant pathogenic strains in the field

**Fig 1:**
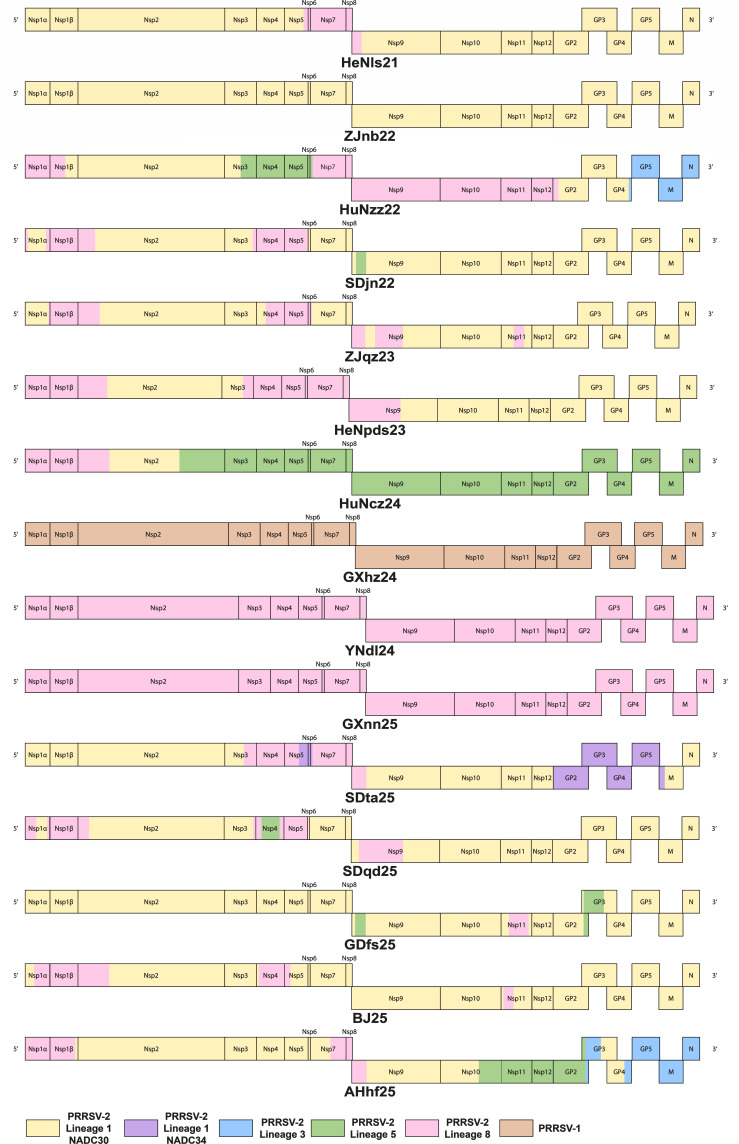
Protein schematic diagram of clinical PRRSV isolates.

To investigate the current epidemiological status of pathogenic PRRSV recombination in China, 15 PRRSV strains isolated from PRRSV outbreak farms between 2021 and 2025 (Table 1) were submitted for whole genome sequencing (WGS), and the sequences were submitted to GenBank. Recombination analysis revealed that 11 out of the 15 isolates (73.3%) possessed recombination signals (Fig. 1). Specifically, strains HeNls21, ZJqz23, HeNpds23, and BJ25 were identified as recombinants between lineage 1 NADC30-like strains and lineage 8 strains. Another group, comprising SDjn22, HuNcz24, SDqd25, and GDfs25, originated from recombination events involving lineage 1 NADC30-like, lineage 8, and lineage 5 strains. More complex recombination patterns were observed in SDta25 (recombinant of lineage 1 NADC30-like, lineage 1 NADC34-like, and lineage 8) as well as HuNzz22 and AHhf25 (recombinants involving lineage 1 NADC30-like, lineage 8, lineage 5, and lineage 3). Notably, all 11 recombinant strains involved genetic exchange between lineage 1 and lineage 8 strains. In terms of structural composition, 6/11 recombinants possessed structural proteins derived entirely from NADC30-like strains, while 4/11 contained partial NADC30-like structural components. Classical phylogenetic analysis based on ORF5 sequences could only classify these strains into lineages; it failed to capture this complex recombination landscape.

**TABLE 1:** Clinical sequences used for recombination analysis in this study

Isolate	GenBank accession no.	Geolocation	Collection year
HeNls21	PX467605	China: Henan	2021
ZJnb22	PX467598	China: Zhejiang	2022
HuNzz22	PX467602	China: Hunan	2022
SDjn22	PX467601	China: Shandong	2022
ZJqz23	PX467597	China: Zhejiang	2023
HeNpds23	PX467604	China: Henan	2023
HuNcz24	PX467603	China: Hunan	2024
GXhz24	PX467607	China: Guangxi	2024
YNdl24	PQ863973	China: Yunnan	2024
GXnn25	PX467606	China: Guangxi	2025
SDta25	PX467599	China: Shandong	2025
SDqd25	PX467600	China: Shandong	2025
GDfs25	PX467608	China: Guangdong	2025
AHhf25	PX467610	China: Anhui	2025
BJ25	PX467609	China: Beijing	2025

Collectively, these findings confirm that NADC30-like recombinants, particularly those involving lineage 8 vaccine donors, currently represent the dominant pathogenic strains circulating in the field.

### NGS reveals the high intrinsic recombination potential of the lineage 5 replicase, which is modulated by nsp9 residue K541

**Fig 2:**
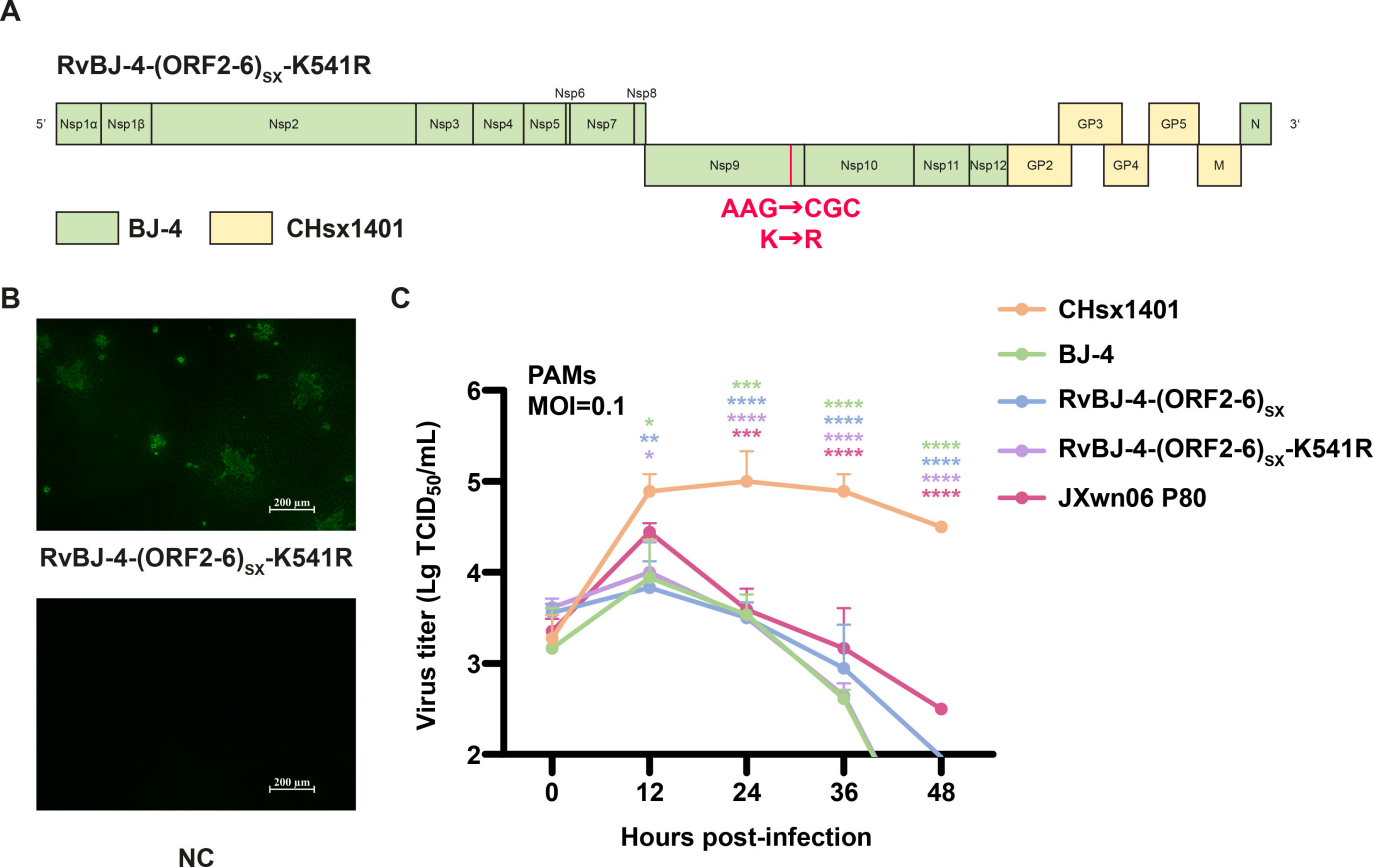
Genomic structure and growth properties of rescued viruses. (**A**) Protein schematic diagram of RvBJ-4-(ORF2-6)_SX_-K541R. (**B**) Identification of rescued virus by IFA. (**C**) Multistep growth kinetics of viruses in PAMs. Statistical significance between CHsx1401 and other strains is indicated by asterisks of different colors corresponding to the strains. *, *P* < 0.05; **, *P* < 0.01; ***, *P* < 0.001; and ****, *P* < 0.0001.

Our previous study identified residue K541 in the RdRP (nsp9) of HP-PRRSV as a key determinant of intrinsic template-switching propensity, and K541R mutation could reduce recombination frequency of JXwn06 (36). To investigate whether this site affects recombination frequency during co-infection, we introduced the K541R mutation into a chimeric virus, RvBJ-4-(ORF2-6)_SX_ (5). This chimera contains the backbone of the lineage 5 strain BJ-4, and substituted structural proteins GP2, GP3, GP4, GP5, and M with NADC30-like strain CHsx1401. The resulting mutant, RvBJ-4-(ORF2-6)_SX_-K541R, mutated residue 541 of nsp9 from lysine (K) to arginine (R) (Fig. 2A), was identified by IFA in MARC-145 (Fig. 2B). Multistep growth kinetics in PAMs showed that all viruses replicated efficiently, although CHsx1401 exhibited significantly higher titers than other strains after 12 hpi (Fig. 2C). It is worth noting that these growth kinetics cannot reflect the situations under co-infection.

**Fig 3:**
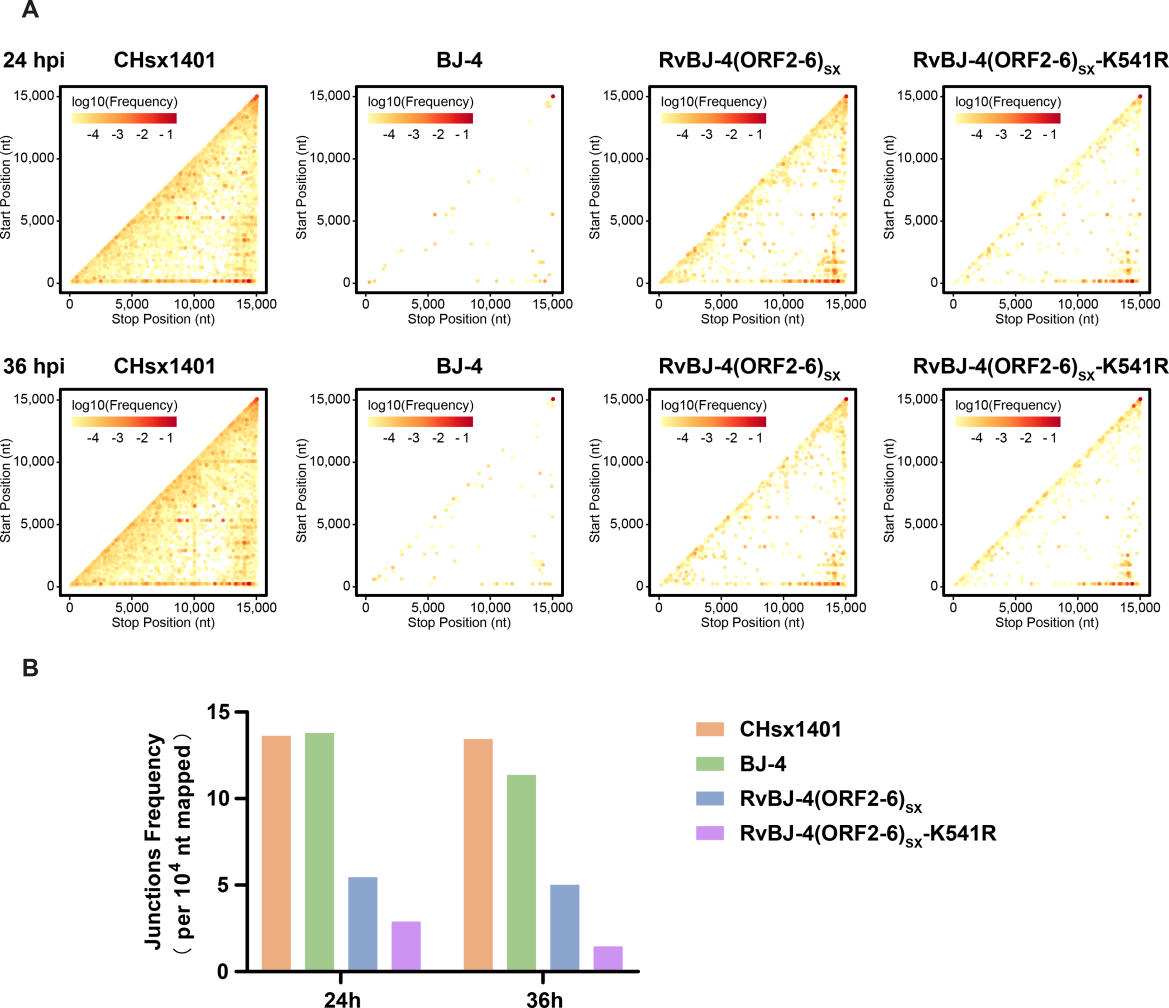
Analysis of intra-strain recombination events. (**A**) Scatter plots of recombination junctions at 24 and 36 hpi. The *x*-axis represents the genomic position of the donor site (5′ end), and the *y*-axis represents the acceptor site (3′ end). Reverse recombination signals were filtered out. Axes represent nucleotide start and stop positions. Off-diagonal signals indicate potential recombination events. Each spot is colored according to its frequency relative to the total population of junctions, transitioning from light yellow (lowest) to deep red (highest). (**B**) Normalized intra-strain recombination junction frequency. Data are expressed as the number of junction reads per 10^4^ mapped nucleotides derived from NGS analysis.

To determine the intrinsic recombination potential of these strains, we analyzed intra-strain recombination (self-recombination) junctions using NGS. PAMs were infected with BJ-4, CHsx1401, RvBJ-4-(ORF2-6)_SX_, or RvBJ-4-(ORF2-6)_SX_-K541R (MOI = 0.1) for 24 or 36 h. Forward (5′→3′) recombination junctions were mapped (Fig. 3A), and normalized to intra-recombination junction frequency (junction reads per 10^4^ mapped nucleotides) (Fig. 3B). Consistent with its status as a highly recombining field strain, CHsx1401 displayed a high frequency of recombination events (13.63 at 24 hpi and 13.44 at 36 hpi). Interestingly, BJ-4 also exhibited a high level of normalized intra-recombination (13.79 at 24 hpi and 11.37 at 36 hpi), comparable to CHsx1401. Despite BJ-4 having a viral titer less than 1/10 of CHsx1401 (Fig. 2C), its replicase generated a similar density of template-switching events per unit of RNA synthesized. This suggests that the polymerase of lineage 5 BJ-4 does not inherently induce low recombination frequency. In contrast, the chimeric strain RvBJ-4-(ORF2-6)_SX_ showed a reduced junction frequency (5.45 at 24 hpi and 5.02 at 36 hpi) compared with its parental backbone BJ-4. Most importantly, the introduction of the K541R mutation further reduced the junction frequency in RvBJ-4-(ORF2-6)_SX_-K541R (2.89 at 24 hpi and 1.46 at 36 hpi), which was less than half that of the non-mutant chimera.

These NGS data indicate that although the BJ-4 backbone replicase is prone to template switching, the chimeric virus exhibits lower junction frequency. Moreover, the K541R mutation effectively reduces recombination events at the nucleic acid level.

### High frequencies of nucleic acid recombination events in lineage 5-based strains do not translate into viable recombinant progeny

The chimeric virus RvBJ-4-(ORF2-6)_SX_ was originally designed as a vaccine candidate against NADC30-like viruses (5). We hypothesized that vaccination might greatly reduce the viremia of homologous NADC30-like strains, so the major concern should be the inter-strain recombination between RvBJ-4-(ORF2-6)_SX_ and other lineage strains. To simulate the common field scenario of recombination with lineage 8 vaccines, JXwn06 P80 (representing lineage eight vaccines) was co-infected with each of the four test strains CHsx1401, BJ-4, RvBJ-4-(ORF2-6)_SX_, or RvBJ-4-(ORF2-6)_SX_-K541R.

**Fig 4:**
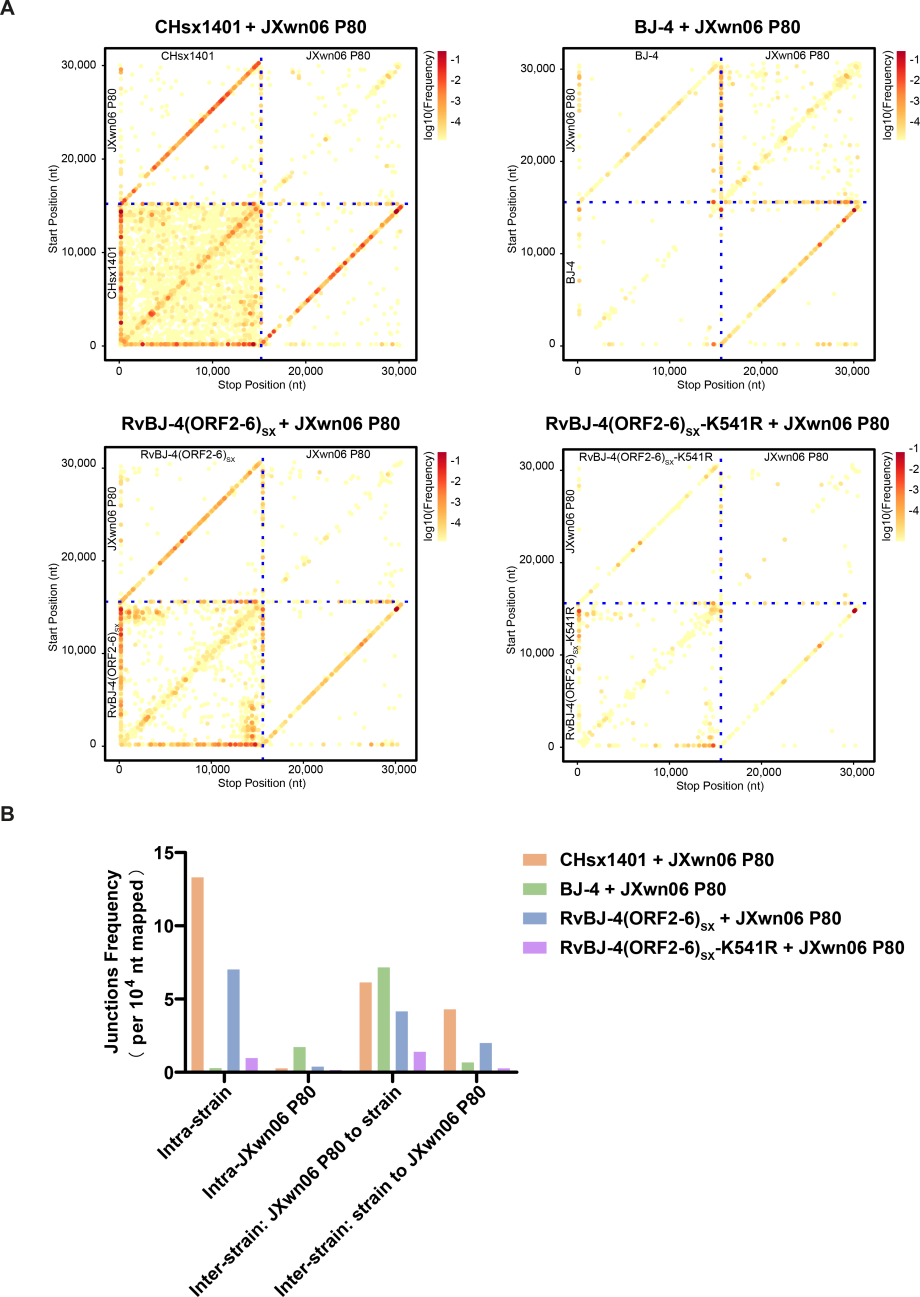
Analysis of recombination during co-infection. (**A**) Scatter plots of recombination junctions at 24 hpi. Axes represent nucleotide start and stop positions on the concatenated reference genome (Test strain + JXwn06 P80). Off-diagonal signals indicate potential recombination events. The blue dashed lines indicate the concatenation junction (split point) between the two viral genomes, dividing the plot into four quadrants that correspond to the four recombination categories. (i) Bottom-left: recombination within the test strain (intra-strain); (ii) top-right: recombination within the JXwn06 P80 strain (intra-JXwn06 P80); (iii) bottom-right: recombination jumping from the test strain to JXwn06 P80 (inter-strain: strain to JXwn06 P80); (iv) top-left: recombination jumping from JXwn06 P80 to the test strain (inter-strain: JXwn06 P80 to strain). The color scale represents the log10 frequency of each unique junction event. (**B**) Normalized recombination junction frequency. Data are expressed as the number of junction reads per 10^4^ mapped nucleotides. The legend (colors) represents the four distinct co-infection experimental groups, while the *x*-axis categories (intra-strain, intra-JXwn06 P80, etc.) directly correspond to the four quadrants defined in the scatter plots of (**A**), where “strain” refers to the specific test virus used in each co-infection group.

After 24 h post co-infection, viral RNAs were extracted, and NGS analysis were performed. Recombination junctions were mapped and visualized in scatter plots (Fig. 4A). The blue dashed lines indicate the concatenation junction between the two viral genomes, dividing the plot into four quadrants: intra-strain (bottom-left), intra-JXwn06 P80 (top-right), and inter-strain recombination events (top-left and bottom-right). In the “BJ-4 + JXwn06 P80” group, the scatter plot displayed a higher density of deep red spots in the top-right quadrant compared with the bottom-left, indicating a higher sequence proportion and replication dominance of JXwn06 P80 over BJ-4. In parallel with the reduced viral abundance, the normalized intra-strain junction frequency of BJ-4 dropped to 0.29 per 10^4^ nt mapped nucleotides (Fig. 4B). In contrast, the “CHsx1401 + JXwn06 P80” group exhibited high frequencies of both intra-strain or inter-strain recombination signals (off-diagonal spots). The normalized data showed that CHsx1401 maintained the highest intra-strain junction frequency (13.31 per 10^4^ nt) even during co-infection. In the “RvBJ-4-(ORF2-6)_SX_ + JXwn06 P80” group, the chimeric virus replicated efficiently (Fig. 4A), and its intra-strain junction frequency reached 7.01 per 10^4^ nt (Fig. 4B), which is comparable to that observed in single-strain infection. As expected, the K541R mutation reduced this frequency in the “RvBJ-4-(ORF2-6)_SX_-K541R + JXwn06 P80” group across all categories: intra-strain (0.96 vs 7.01), intra-JXwn06 P80 (0.15 vs 0.39), and inter-strain junctions. These results confirm that K541R effectively reduces recombination potential at the molecular level.

**Fig 5:**
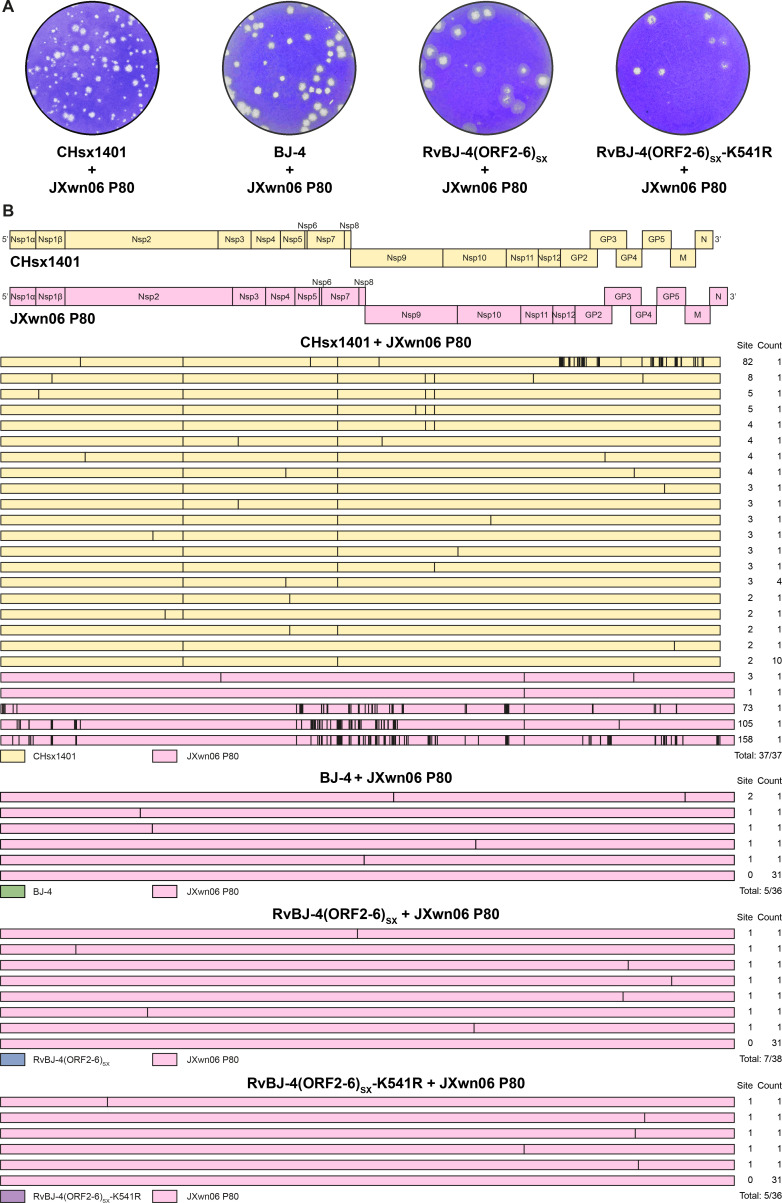
Plaque purification and genomic characterization of progeny viruses from JXwn06 P80 co-infection groups. (**A**) Plaque morphology observed by crystal violet staining. (**B**) Schematic of recombination breakpoints in plaque-purified viruses. Each horizontal bar represents a kind of viral genome. The background color indicates the major parental backbone, while the black vertical lines (|) indicate the precise recombination sites containing sequences derived from the co-infecting strain. “Site” indicates the total number of recombination breakpoints in a single sequence. Note: Due to scaling, some adjacent sites may appear merged; the exact count is provided in the “site” column. “Count” indicates the number of isolates exhibiting this specific recombination pattern. “Total” represents the proportion of recombinant sequences relative to the total number of viable viruses sequenced.

To determine which recombinant genomes could assemble into infectious particles and survive host selection, plaque purification was performed. After 24 h of co-infection, viral titers were measured. The titer of the “RvBJ-4-(ORF2-6)_SX_-K541R + JXwn06 P80” group (5.33 ± 0.00 Lg TCID_50_/mL) was lower than that of the CHsx1401 (6.50 ± 0.00 Lg TCID_50_/mL), BJ-4 (6.22 ± 0.63 Lg TCID_50_/mL), and RvBJ-4-(ORF2-6)_SX_ (5.89 ± 0.19 Lg TCID_50_/mL) co-infection groups. Crystal violet staining revealed two distinct plaque sizes within single groups (Fig. 5A), potentially indicating phenotypic diversity among recombinants, although no direct correlation between plaque size and specific recombination sites was established. Out of 40 picked plaques per group, 36 to 38 viable isolates were sequenced. Recombination analysis (Fig. 5B) showed that 100.0% (37/37) of isolates were recombinants in the “CHsx1401 + JXwn06 P80” group. Notably, 32 of these utilized CHsx1401 as the major parental backbone, often with shared recombination breakpoints in nsp2 and nsp7, suggesting adaptive selection for these specific crossover events. In stark contrast, despite the high junction frequency observed in NGS for the RvBJ-4-(ORF2-6)_SX_ group, strains utilizing the lineage 5 BJ-4 backbone (BJ-4, RvBJ-4-(ORF2-6)_SX_, and K541R mutant) yielded a low proportion of recombinant plaques (13.9%, 18.4%, and 13.9%, respectively) (Fig. 5B). Crucially, unlike the CHsx1401 group, all recombinant isolates in these groups utilized JXwn06 P80 as the major parental backbone. This suggests that while lineage 5-based replicases (including the Chimera) generated recombination events physically, progeny viruses carrying the lineage 5 backbone were largely eliminated by competitive selection.

Furthermore, the K541R mutation did not further reduce the proportion of viable recombinants (5/36, 13.9% vs 7/38, 18.4%), indicating that under the strong selective pressure exerted by JXwn06 P80, the bottleneck for recombinant survival is determined by backbone fitness rather than recombination frequency alone.

### Co-infection with NADC30-like strains promotes the emergence of viable recombinants predominantly utilizing the lineage 1 backbone

**Fig 6:**
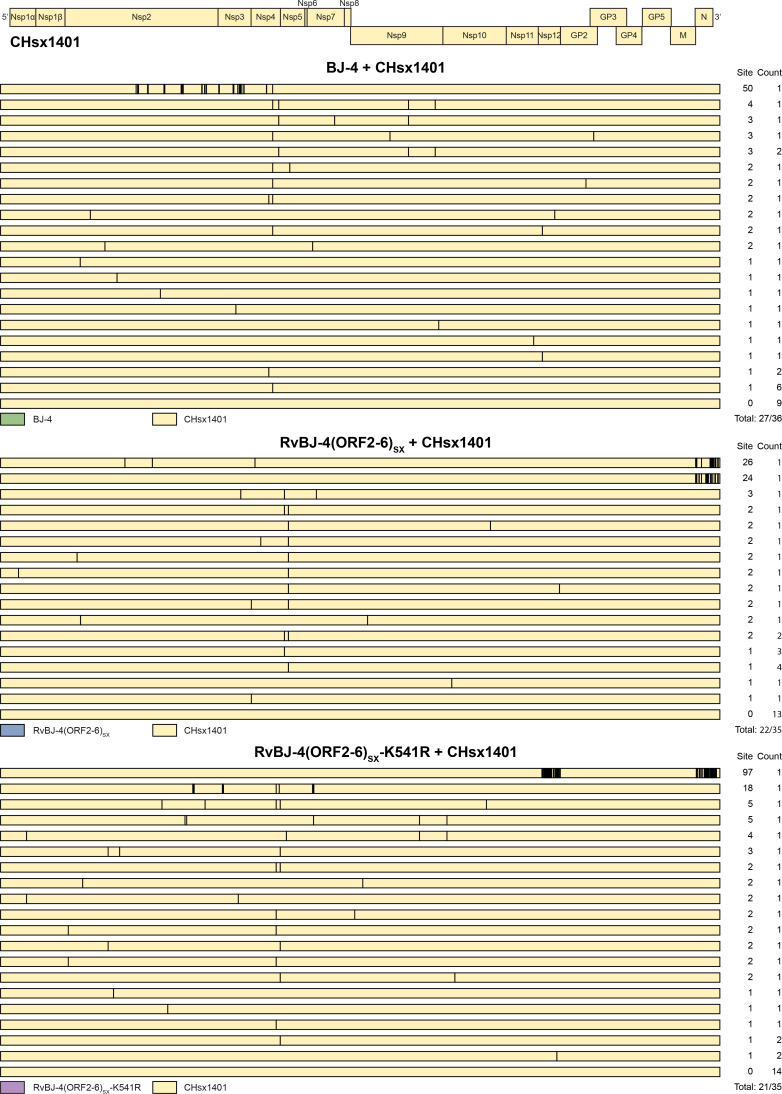
Genomic characterization of progeny viruses from CHsx1401 co-infection groups. Each horizontal bar represents a kind of viral genome. The background color indicates the major parental backbone, while the black vertical lines (|) indicate the precise recombination sites containing sequences derived from the co-infecting strain. “Site” indicates the total number of recombination breakpoints in a single sequence. Note: Due to scaling, some adjacent sites may appear merged; the exact count is provided in the “Site” column. Count” indicates the number of isolates exhibiting this specific recombination pattern. “Total” represents the proportion of recombinant sequences relative to the total number of viable viruses sequenced.

**Fig 7:**
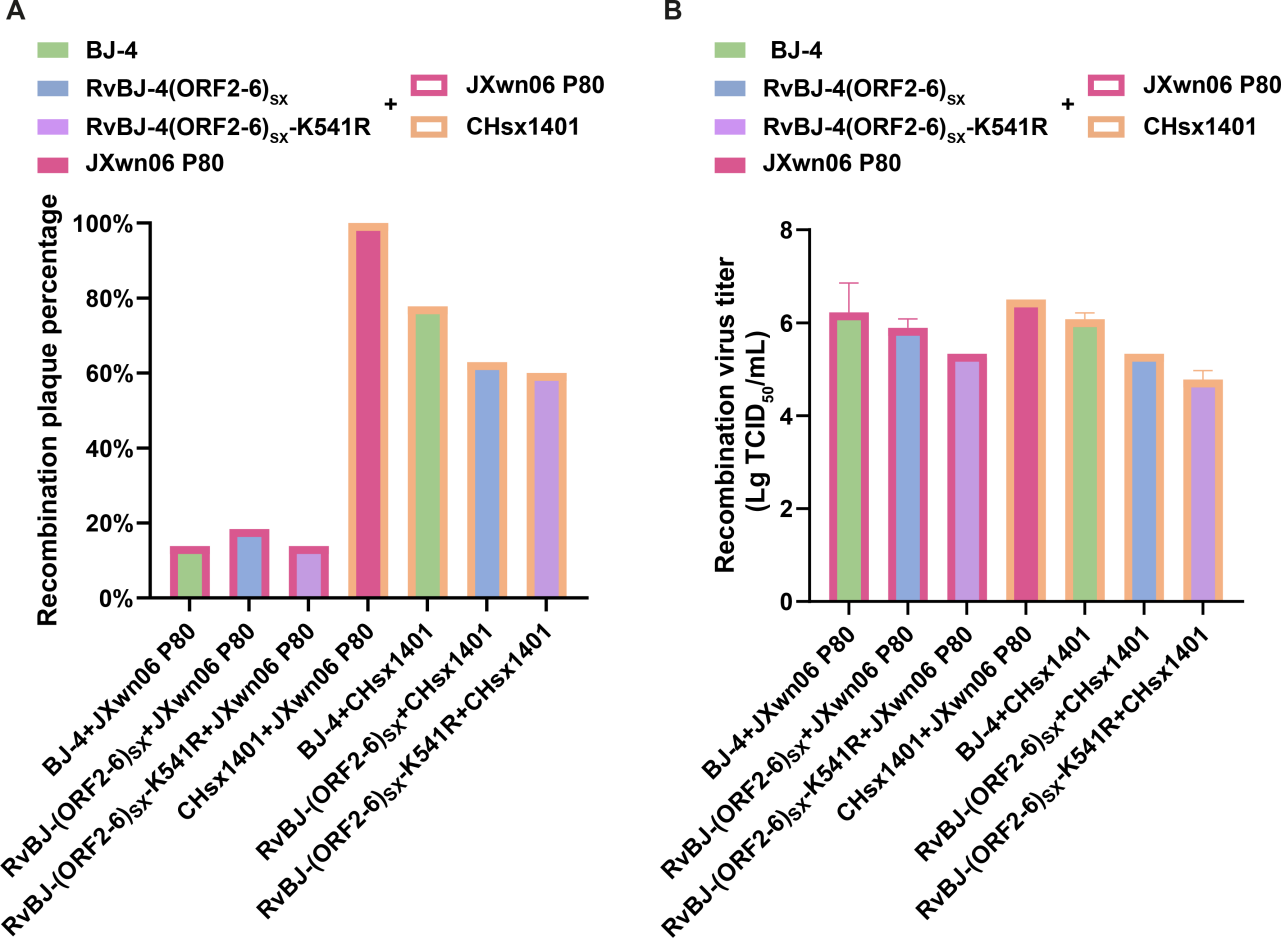
Summary of recombination outcomes. (**A**) Percentage of recombinant plaques identified in each co-infection group. (**B**) Viral titers of the co-infection culture supernatants. The colors of the column fill and border correspond to the two strains involved in the co-infection. Note: statistical comparison markers are omitted for clarity due to the large number of comparison groups.

Field epidemiology suggests that NADC30-like strains act as a “reservoir” for recombination. Experiments above indicate that co-infection between BJ-4 and JXwn06 P80 produce relatively small amounts of recombinants, while CHsx1401 produce 100% recombinants in case of co-infection with JXwn06 P80. To experimentally verify the pivotal role of NADC30-like strains in this process, we performed a reciprocal co-infection assay. Instead of the lineage eight vaccine-candidate strain, we utilized the lineage 1 NADC30-like strain CHsx1401 as the co-infection strain for BJ-4, RvBJ-4-(ORF2-6)_SX_, and the K541R mutant. Plaque purification and WGS revealed that the proportion of recombinant progeny increased in all groups containing CHsx1401 compared with those co-infected with JXwn06 P80. Specifically, the recombination rates rose to 75.0% (27/36) for BJ-4, 62.9% (22/35) for RvBJ-4-(ORF2-6)_SX_, and 60.0% (21/35) for the K541R mutant (Fig. 6) compared with less than 20% in the lineage 8 co-infection groups. In short, the recombination plaque percentage consistently remains high whenever CHsx1401 is involved in the co-infection process (Fig. 7A, yellow-bordered columns). Notably, BJ-4 was less prone to generating viable recombinants than JXwn06 P80 when both were co-infected with CHsx1401 (75.0% vs 100%). Sequence analysis showed that all of the identified recombinants in these groups utilized CHsx1401 as the major parental backbone (Fig. 6). This mirrors the findings from the JXwn06 P80 co-infection experiments, where the lineage 5 backbone was consistently eliminated. Moreover, complex continuous fragments recombination signals were detected in progeny viruses whenever CHsx1401 was involved in co-infection, including combinations with JXwn06 P80 (Fig. 5B) and BJ-4 strains (Fig. 6).

Consistent with previous results, the K541R mutation failed to provide a significant reduction in recombination frequency in this context (60.0% for K541R mutant vs 62.9% for RvBJ-4-(ORF2-6)_SX_). Although the proportion of recombinants was higher, the total viral titers in the CHsx1401 co-infection groups were generally lower than those observed in the JXwn06 P80 co-infection groups, while this difference was not statistically significant (Fig. 7B).

In summary, our data indicate that NADC30-like strains play a pivotal role in driving PRRSV recombination. Whenever CHsx1401 is involved, the frequency of viable recombinant progeny remains high, and the strong selective pressure favors the survival of the NADC30-like backbone over the lineage 5 backbone.

## DISCUSSION

With the rapid development of WGS technology, the complex landscape of PRRSV recombination has become increasingly transparent. Our clinical sequence data, derived from outbreak farms, mirror recent large-scale surveillance studies (20, 27), confirming that recombinant strains between lineage 1 NADC30-like and lineage 8 HP-PRRSV vaccine strains have become the predominant pathogenic strains in China. Moreover, due to the prohibitive cost of WGS in the past, a significant number of isolates were not subjected to full-length sequencing and recombination analysis. Consequently, most strains were classified as lineage 1 NADC30-like based solely on ORF5 gene sequences. This limited historical data suggest that the timeframe when recombinant strains achieved dominant prevalence in China may have been earlier than currently documented. In contrast, although lineage 5 classical vaccine strains are widely used, and establish a ubiquitous baseline presence in vaccinated herds (representing massive opportunities for co-infection with wild-type strains), viable recombinants harboring the lineage 5 backbone are infrequently detected in the field. While subclinical circulation of such recombinants cannot be completely ruled out due to sampling biases, their failure to emerge as dominant pathogenic strains—unlike recombinants derived from lineage 8 vaccines—highlights a profound competitive disadvantage. Previously, this was often attributed to a presumed high replication accuracy of lineage 5 strains. However, our study provides a new perspective: the scarcity of lineage 5 recombinants is not driven by intrinsic template-switching propensity, but by the low evolutionary fitness and competitive exclusion of their progeny.

To elucidate the mechanisms driving these evolutionary patterns, we utilized a co-infection model with an MOI of 0.5 + 0.5. This design was chosen to simulate a complex natural PRRSV co-infection status, characterized by a proportion of susceptible macrophages simultaneously infected by two strains alongside remaining singly infected cells. The detection of recombinants in this situation indicates that template switching and genetic exchange can occur even when dual-infected cells are not maximized experimentally. In other words, our data suggest that PRRSV recombination does not strictly require artificially saturated infection conditions and may indeed arise under more realistic field-like scenarios where multiple variants co-circulate but do not infect every cell at the same time. Despite this favorable environment for genetic exchange, a key finding of this study is the disconnect between recombination events at the nucleic acid level and the survival of recombinant viable virus particles. Our NGS data revealed that the lineage five replicase generated normalized junction frequencies comparable to, or even higher than, those of NADC30-like strains when viral RNA abundance was high. However, these recombination events rarely translate into viable progeny in a co-infection setting. We postulate that this failure is driven by the evolutionary status of lineage 5 strains. The replication efficiency, viral productivity, replicase accuracy and other properties of strains affect the clinical feature of prevalence and recombination. The inter-recombination requires two or more viruses entering the same host cell. Lower replication efficiency results in fewer viral particles and a short period of viremia *in vivo* (5, 39), therefore reducing the probability of two particles infecting the same cell. Unlike NADC30-like or HP-PRRSV strains, which have accumulated mutations to optimize replication and evade host immunity, lineage 5 strains retain many “ancestral” genomic features and lack key virulence-enhancing and replicase-enhancing mutations in non-structural proteins (21, 22). Consequently, even if a lineage 5 strain successfully acquires a foreign gene segment via recombination, the lineage 5 backbone itself functions as an evolutionary bottleneck. Within the context of our findings, this evolutionary bottleneck is defined not by the information decay threshold, but as a scenario where the phenotypic deficits of a specific viral backbone, such as suboptimal replication efficiency and vulnerability to host clearance, lead to the competitive exclusion of its recombinant progeny. The resulting recombinant is likely to be targeted by the host or outcompeted by the parental strains, leading to its elimination. This hypothesis aligns with our field observation that surviving pathogenic recombinants almost exclusively utilized the highly fit NADC30-like or HP-PRRSV backbones, while lineage 5-based recombinants were essentially eliminated by the selection pressure. This suggests that the fate of lineage 5-based recombinants might be limited by their specific phenotypic deficits, which likely extend beyond low replication efficiency and host clearance to include severe intermediate incompatibilities, such as structural protein mismatches and packaging failures.

In stark contrast, NADC30-like strains (CHsx1401) exhibited a dominant role in driving recombination. Whether co-infected with lineage 8 or lineage 5 strains, groups involving CHsx1401 consistently yielded the highest proportion of recombinant plaques. This can be attributed to two factors. First, their robust replication in PAMs provides a high abundance of RNA templates, physically increasing the probability of template switching. Second, and perhaps more importantly, the NADC30-like genome appears to facilitate or tolerate complex genomic mosaicism. In our study, complex “continuous fragment recombination” signals were frequently detected in groups involving CHsx1401, which is the same as that of the clinical isolates. Crucially, this phenomenon was not limited to the CHsx1401 backbone; even recombinants utilizing the JXwn06 P80 backbone displayed complex mosaic patterns when CHsx1401 was present. This implies that the presence of NADC30-like strains creates a cellular environment highly conducive to genetic exchange, acting as a highly recombinogenic backbone that fuels viral evolution. Thus, the NADC30-like strain exemplifies a phenotypic profile perfectly suited for recombination: high replicative capacity to provide templates, low replication accuracy to generate crossovers, and robust fitness to ensure progeny survival.

The hinge role of NADC30-like strains in field recombinants with HP-PRRSV MLV strains poses a considerable concern for vaccine safety. Vaccines derived from lineage 8 HP-PRRSV strains have caused significant safety issues. Clinical experiments have verified that JXwn06 P80 could revert to mortality only after the ninth passage in pigs (40), and revertants of JXA1 P80 (also known as JXA1-R) have been detected in the field from 2015 (24) up to recent years (25). In another study, a target sequence of microRNA-142 (miR-142) was inserted into JXwn06 to inhibit viral replication in PAMs that highly expresses miR-142, while the virus isolated from serum was observed to have partial deletion of the miR-142 target sequence at 14 dpi, and reverted to virulence, causing mortality only after 19 days post-inoculation (dpi) (41). The use of such genetically unstable vaccines is a major safety concern. As for the prevalence NADC30-like strain, vaccines derived from other lineages provide limited cross-protection against it (39). In this context, our chimeric vaccine candidate RvBJ-4-(ORF2-6)_SX_ has shown a favorable balance between safety and efficacy (5). Although the lineage 5 replicase is prone to errors, the low fitness of its backbone may paradoxically serve as a safety feature, as recombinant progeny carrying this backbone are less likely to survive in the field. It is worth noting that the recombination plaque rate of BJ-4 or BJ-4 backbones was notably reduced compared with CHsx1401 or JXwn06 P80. Conversely, utilizing passaged-attenuated NADC30-like strains as vaccines would pose a significantly higher risk of recombination and reversion to virulence, as they lack this evolutionary barrier and inherently drive genomic exchange. However, the risk remains that lineage 5 vaccines could serve as gene donors to NADC30-like strains, contributing to the diversity of the recombinogenic backbone. Therefore, monitoring the phenotypic compatibility of vaccine backbones with circulating field strains is as crucial as monitoring their genetic stability. Besides, rewiring TRS to create mismatch, thereby inhibiting the production of sgRNA, might be a promising way to prevent recombination (27).

The K541R mutation, which was previously identified as a key recombination frequency determinant, was evaluated as a potential strategy to improve vaccine safety. As expected, K541R successfully reduced recombination junctions at the nucleic acid level (NGS), but the reduced proportion of viable recombinant progeny in co-infection plaques is limited. This highlights a critical limitation: replicase accuracy is only one part of the equation. Recombination also depends on template availability, replication efficiency, viral productivity, and superinfection dynamics (37, 38). In our model, the reduced error rate provided by K541R could not overcome the overwhelming selective pressure exerted by highly replicative field strains. To further validate this function, we also constructed a CHsx1401-K541R mutant and subjected it to the same series of co-infection, plaque purification, and WGS experiments with JXwn06 P80, RvBJ-4-(ORF2-6)_SX_, and RvBJ-4-(ORF2-6)_SX_-K541R (data not shown). Analysis of the isolates consistently revealed that all resulting recombinants utilized CHsx1401 as the backbone, exhibiting multiple signals of “continuous fragment recombination” in several plaques, and the recombination rate remained unchanged even in the co-infection group involving the double replicase mutants, RvBJ-4-(ORF2-6)_SX_-K541R and CHsx1401-K541R (data not shown). These results underscore that modifying a single trait is insufficient when the viral backbone inherits a complex mix of weaknesses or strengths that complicate its competitive outcome in a complex cellular environment.

There are several limitations in this study that should be noted. First, the transition from co-infection in primary PAMs to plaque purification in MARC-145 cells imposes a specific selective pressure (host switching) that may not fully replicate the complex environment of the natural host. For instance, the MARC-145-passaged JXwn06 P80 strain may be better adapted to this cell line, leading to robust growth. We could not rule out mutations or selection bias introduced during propagation in MARC-145 cells. Second, due to the labor-intensive process of plaque picking and individual WGS, the plaque purification experiment was performed once without biological replicates for statistical analysis of the plaque proportions. However, the consistent trends observed across multiple groups and their alignment with the NGS data support the validity of our conclusions. Finally, our understanding is limited by the lack of comprehensive phenotypic data across different PRRSV strains. As demonstrated in this study, the complexity of PRRSV recombination stems from distinct, multi-dimensional phenotypic profiles, involving a complex interplay of replication efficiency, viral productivity, replicase accuracy, immune evasion, and competitive fitness. However, we do not yet know the full landscape of these traits for all field strains, nor is it clear if they are consistent within a lineage. The unpredictable phenotypic profile of novel recombinants adds another layer of complexity, making the full characterization of PRRSV recombination dynamics a significant challenge.

### Conclusion

In conclusion, this study reveals that PRRSV recombination is a multi-factorial process governed by the distinct phenotypic profiles of interacting strains. We demonstrate that the widespread prevalence of NADC30-like recombinants is driven by the strain’s unique combination of robust replication capacity and tolerance for genomic mosaicism. Conversely, the infrequency of lineage 5 recombinants stems from the competitive exclusion of lineage 5 backbones due to their specific phenotypic deficits, including low replication efficiency and weak resistance to host selection, rather than low recombination frequency. These insights suggest that future research and vaccine design must consider the comprehensive landscape of viral phenotypes, encompassing replication accuracy, fitness, and competitiveness, to effectively predict and mitigate recombination risks.

## MATERIALS AND METHODS

### Clinical PRRSV sequences

PRRSV strains listed in Table 1 were isolated from PRRSV-positive field samples collected in China between 2021 and 2025, sequenced, and previously submitted to GenBank. These sequences were used for recombination analysis.

### Recombination analysis of field and plaque-purified PRRSV sequences

PRRSV sequences for recombination analysis were aligned in Geneious Prime (version 2025.0.2) with representative strain sequences. For clinical PRRSV sequences, recombination analysis was conducted using Simplot (version 3.5.1) with default settings. For co-infected followed by plaque purification samples, a nucleotide position at which the sequence switched from the major backbone strain to the co-infecting strain was defined as a recombination site, and the recombination sites were numbered and marked in Adobe Illustrator to construct a length-proportional protein schematic diagram.

### Cell lines, plasmids, and viruses

Primary porcine alveolar macrophages (PAMs) were prepared and cryopreserved from 4-week-old specific-pathogen-free (SPF) landrace pigs as previously described (42). PAMs were thawed and cultured in RPMI 1640 medium (#61870044, Gibco, USA) with 10% FBS (#16140071, Gibco, USA) and penicillin (50 U/mL)-streptomycin (50 mg/mL) at 37℃ with 5% CO_2_, and replaced with 2% 1640 medium after infection. MARC-145 cells were cultured in Dulbecco’s modified Eagle’s medium (DMEM) (#12491015, Gibco, USA) with 10% FBS and penicillin (50 U/mL)-streptomycin (50 μg/mL) at 37℃ with 5% CO_2_ and replaced with 2% DMEM medium after infection.

The plasmids of the full-length infectious clone of RvBJ-4-(ORF2-6)_SX_ were described previously (5). RvBJ-4-(ORF2-6)_SX_-K541R was constructed by site-directed mutagenesis based on RvBJ-4-(ORF2-6)_SX_. Specifically, the full-length plasmid of RvBJ-4-(ORF2-6)_SX_ was amplified using KOD One PCR Master Mix (#KMM-201, TOYOBO, Japan) with a pair of partially complementary primers BS-K541R-F (CCA**CGC**AAGACAGCAATAACAGACTCGCCATC) (bolded bases represent the arginine) and BS-K541R-R (ATTGCTGTCTT**GCG**TGGGTCCGTCTGAAACCCC) (bolded bases represent the arginine), followed by digestion with DpnI (#R0176, NEB, USA) for an hour, and purified using an Ultra-Sep Gel Extraction Kit (#D2510, OMEGA, USA). The purified product was applied to homologous recombination using a ClonExpress Ultra One Step Cloning Kit (#C112-02, Vazyme, China), followed by transformation of the recombinant plasmid, screening, propagation, and plasmid extraction (#A2492, Promega, USA). The mutant viruses were rescued in HEK-293T cells, as previously described (5). Rescued viruses were confirmed by an indirect immunofluorescence assay (IFA) using PRRSV N protein-specific monoclonal antibodies (prepared and stored in our laboratory).

Viruses CHsx1401 (represent lineage 1 NADC30-like strain), BJ-4 (represent lineage five classical strain), and JXwn06 P80 (JXwn06 passaged in MARC-145 cells for 80 times, represented lineage 8 vaccines) were propagated and stored in our laboratory. To analyze the *in vitro* growth characteristics, virus titers in cell cultures were determined by a microtitration infectivity assay and recorded as 50% tissue culture infective dose per milliliter (TCID_50_/mL) using the Reed-Muench method in MARC-145 cells, with a detection limit of 10^2^ TCID_50_/mL.

### Infection, Co-infection, and NGS

After thawing and culturing PAMs for 6 h, viruses were pre-mixed with 2% 1640 medium to MOI = 0.1 for single-strain infection, or MOI = 0.5 for each virus (total MOI = 1.0) for two-strain co-infection, and incubated with PAMs for 1 h at 37℃ 5% CO_2_. After the inoculation phase, the inoculum was removed, and PAMs were further cultured in 2% 1640 medium. After 24 or 36 h post-inoculation (hpi), PAMs were frozen and thawed from −80℃ to room temperature, and cell debris was removed by low-temperature high-speed centrifugation to obtain the co-infection virus suspensions. RNA was extracted by MagZol Reagent (#R4801, Magen, China) and sent to BIOYIGENE Biotechnology (Wuhan, China) (for single strain infection) or Sangon Biotechnology (Shanghai, China) (for two-strain co-infection) for NGS. Illumina RNA-Seq processing, alignment, and recombination junction analysis were conducted as previously described (35, 43).

### Plaque purification and WGS

For the plaque purification assay, MARC-145 cells in six-well plates were infected with the suspensions from virus co-infected PAMs with gradient dilution, and incubated for 1 h at 37℃ with 5% CO_2_. After the inoculation phase, the inoculum was removed, 2% low melting point agarose (#A8350, Solarbio, China), 2× DMEM, and 4% FBS were premixed and added onto the cells. After solidification at room temperature, the plate was inverted and cultured until viral plaques appeared. The gel was disposed of, and 2 mL of 4% formaldehyde was added to the well, and the shape of plaques was observed by staining with a 5% crystal violet solution (#C0121, Beyotime, China). In parallel, 40 plaques on a separate plate were picked and passaged onto fresh MARC-145 cells to obtain the plaque-purified viruses.

The whole genomes of all viable plaque viruses were sequenced by WGS and compared with parental viruses. In detail, viral RNA from plaques was extracted by Virus DNA/RNA Extraction Kit 2.0 (Vazyme, RM401), followed by reverse transcription with First-strand cDNA Synthesis Mix (#F0202, LabLead, China). For each plaque virus, the KOD One PCR Master Mix was used for PCR amplification with 5 pairs of primers (Table 2), respectively. The five amplified products were mixed into one tube and sent for sequencing by Fast NGS (Tsingke Biotechnology, Beijing, China). The WGS method has been verified and compared with Sanger sequencing to ensure the accuracy of the sequencing. The sequencing results were assembled and compared with that of the parental virus in Geneious Prime.

**TABLE 2:** Primers used for WGS in this study

Primer	Sequence (5′−3′)	Position^*a*^	Length^*a*^
U1F	ATGACGTATAGGTGTTGGCTCT	1–22	3,270
U1R	GTCRATRATGGCTTGAGCYGAG	3,249–3,270	
U2F	ATYACRCGCCCRAARTACTC	3,232–3,251	3,040
U2R	GGAGRAARAAYACACAMAGAAG	6,250–6,271	
U3F	TGCTTGCTGCCAAACCYGARCT	6,203–6,224	2,404
U3R	RAYGCTCTGAATGTCCTTGGTT	6,203–8,606	
U4F	ACAAGGTTTGGRGACATACCTT	8,172–8,193	3,890
U4R	CCYAAMGTTGGTTCAAYGAY	12,042–12,061	
U5F	TYGAATCGGAYACAGCGTAY	11,896–11,915	3,516
U5R	AATTTCGGCCGCATGGTTCTCGCCAATTAAA	11,896–15,411	

^*a*^ Nucleotide positions and amplification lengths are relative to the VR-2332 strain (GenBank accession no. U87392).

### Statistical analysis

Data were expressed as means ± standard deviations. The significance of the variability was determined by two-way ANOVA multiple comparisons using GraphPad Prism (version 10.4.0). Asterisks indicate statistical significance; NS, no significance; *, *P* < 0.05; **, *P* < 0.01; ***, *P* < 0.001; and ****, *P* < 0.0001.

## Data Availability

The complete genome sequences of the clinical PRRSV isolates analyzed in this study have been deposited in the NCBI GenBank database under accession numbers PX467597 to PX467610 and PQ863973.
